# Glyphosate resistance as a potential driver for the dissemination of multidrug-resistant clinical strains

**DOI:** 10.3389/fmicb.2026.1740431

**Published:** 2026-03-24

**Authors:** Camila A. Knecht, Barbara Prack McCormick, Verónica E. Álvarez, Adrián Gonzales Machuca, Fernanda Buzzola, Julio Fuchs, Pablo Salgado, Josefina Campos, Jochen A. Müller, María Paula Quiroga, Daniela Centrón

**Affiliations:** 1 Departamento de Microbiología, Parasitología e Inmunología, Facultad de Medicina. Universidad de Buenos Aires, Buenos Aires, Argentina; 2 Laboratorio de Investigaciones en Mecanismos de Resistencia a Antibióticos (LIMRA), Instituto de Investigaciones en Microbiología y Parasitología Médica (IMPaM), CONICET - Universidad de Buenos Aires, Buenos Aires, Argentina; 3 Facultad de Ciencias Agrarias, Universidad de Lomas de Zamora, Lomas De Zamora, Argentina; 4 Laboratorio de Patogenia Bacteriana (PatoBac), Instituto de Investigaciones en Microbiología y Parasitología Médica (IMPaM), CONICET - Universidad de Buenos Aires, Buenos Aires, Argentina; 5 División de Investigaciones Toxicológicas (DEITOX), Departamento de Bioseguridad y Toxicologia (DEIBIOTOX), Instituto de Investigaciones Científicas y Técnicas para la Defensa (CITEDEF), Buenos Aires, Argentina; 6 Instituto de Investigaciones en Salud Pública, Facultad de Odontología, Universidad de Buenos Aires, Buenos Aires, Argentina; 7 Plataforma de Genómica y Bioinformática, INEI-ANLIS “Dr. Carlos G. Malbrán”, Buenos Aires, Argentina; 8 Unit Head Emerging Pathogen Intelligence Platforms Unit, World Health Organization, Geneva, Switzerland; 9 Institute for Biological Interfaces (IBG-5), Karlsruhe Institute of Technology, Eggenstein-Leopoldshafen, Germany; 10 Clúster de Bioinformática, Instituto de Investigaciones en Microbiología y Parasitología Médica (IMPaM), CONICET - Universidad de Buenos Aires, Buenos Aires, Argentina

**Keywords:** antimicrobial resistance, Argentina, efflux pumps, EPSPs, glyphosate, herbicides, one health

## Abstract

The rise of antimicrobial resistance (AMR) constitutes a serious threat to global health. Environmental bacterial communities are a key reservoir of AMR genes (ARGs) that can spread to clinical pathogens. Biocides, which include broad-spectrum herbicides, can co-select for ARGs, posing a potential driver for AMR spread. Glyphosate, the world’s most widely used herbicide with known bactericidal properties, targets the shikimate pathway and may thus exert selective pressure favoring resistant bacteria, potentially elevating clinical AMR risk from a One Health perspective. We assessed glyphosate resistance in multidrug-resistant (MDR) species isolated from nosocomial infections. Furthermore, we investigated the relationship between glyphosate-resistant environmental species and clinically relevant MDR pathogens using whole-genome sequencing of environmental and clinical strains. Multidrug-resistant species from hospital-acquired infections exhibited high levels of glyphosate resistance. We established a link between glyphosate-resistant environmental species and typically MDR species common in nosocomial settings. Genomic analysis revealed that glyphosate resistance is partially independent of mutations in the target enzyme (5-enolpyruvylshikimate-3-phosphate synthase), suggesting the contribution of alternative mechanisms, such as efflux pumps. Our findings indicate that glyphosate exposure could favor the prevalence of bacteria associated with nosocomial infections and the rise of MDR clinical strains. This suggests that intensive glyphosate use may accelerate the dissemination of AMR. Consequently, the AMR dimension should be incorporated into the environmental risk assessment of biocidal products that are not used as antimicrobial agents.

## 1 Introduction

Multidrug-resistant (MDR) bacteria pose one of the most serious global health threats (World Health Organization, 2021). Although clinical antibiotic use is a well-established driver of antimicrobial resistance (AMR; Fanelli et al., 2020), the environmental drivers of AMR and their connection to human and animal health under the One Health framework are not fully understood (Larsson and Flach, 2021; Li et al., 2025). Evidence suggests a growing link between environmental and clinical resistomes, with soil antimicrobial resistance genes (ARGs) increasing in abundance and mobility, and correlating with trends in clinical resistance (Zhao et al., 2025). However, the specific anthropogenic agents responsible for this connectivity remain poorly characterized.

In contrast to antibiotics, which are not typically applied directly to agricultural soils, herbicides are extensively applied and may exert unintended selective pressure on soil bacterial communities (Banerjee and van der Heijden, 2022). Glyphosate, the active ingredient of the most widely used herbicide worldwide, inhibits the shikimate pathway (Myers et al., 2016). In Argentina, estimated annual glyphosate use averaged 36.278 ± 4.523 tons between 2020 and 2023 (National Registry of Plant Therapeutics of SENASA, 2023), predominantly in soybean cultivation (Aparicio and De Gerónimo, 2024) and peri-urban horticulture (Mac Loughlin et al., 2022).

**Figure 1:**
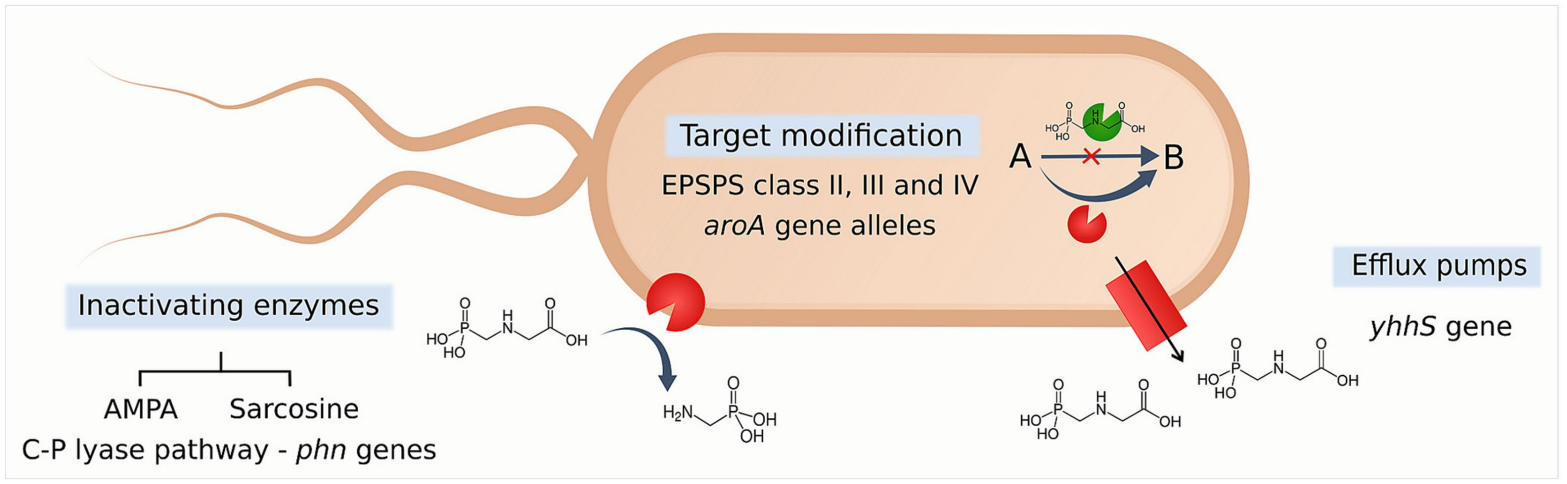
Multifactorial mechanisms of bacterial resistance to glyphosate. Glyphosate resistance can be achieved by target modification (EPSPS coded by different alleles of *aroA*), inactivating enzymes (sarcosine and AMPA pathways, *phn* genes), and efflux pumps (*mexAB* and *acrAB* genes). EPSPS, 5-enolpyruvyl-shikimate-3-phosphate synthase; AMPA, *α*-amino-3-hydroxy-5-methyl-4-isoxazolepropionic acid. The figure was created using Inkscape.

Glyphosate targets the shikimate pathway, which is absent in animals but present in plants, fungi, and bacteria (Hertel et al., 2021). Resistance to glyphosate in bacteria can be achieved by several mechanisms (Figure 1). The most extensively explored mechanism involves the target-site modification of the enzyme 5-enolpyruvylshikimate-3-phosphate synthase (EPSPS), encoded by the *aroA* gene (Funke et al., 2006). Four EPSPS classes have been identified, with class I being susceptible to glyphosate and classes II–IV considered resistant (Leino et al., 2021). Additional resistance mechanisms include efflux pumps and enzymatic inactivation of glyphosate via the sarcosine or AMPA pathways, both of which rely on the C–P lyase system encoded by widely distributed *phn* operons (Hove-Jensen et al., 2014; Hertel et al., 2021).

While herbicides have long been in use, their potential role in driving AMR has only recently gained attention (Bote et al., 2019; Liao et al., 2021; Zerrouki et al., 2024). This connection is plausible because exposure to a single selective agent, like glyphosate, can enrich for bacteria with an MDR phenotype through co-resistance (linked resistance genes) or cross-resistance (shared resistance mechanisms; Paul et al., 2019). Glyphosate exposure has been linked to mutations in regulatory regions of multidrug efflux pumps (Liao et al., 2021) and to an increased abundance of efflux pump genes in water mesocosms (da Barbosa Costa et al., 2022). Given that opportunistic human pathogens, including MDR strains, can persist in soil (Berg et al., 2005), the widespread use of glyphosate in agriculture may favor the selection of clinically relevant resistant bacteria.

Current evaluations of herbicide-impacted soil resistomes primarily rely on culture-independent methods such as qPCR and shotgun metagenomics (Ferreira et al., 2023). While powerful for profiling genetic potential, these approaches cannot confirm functional resistance or capture dynamic physiological responses. In contrast, culture-dependent phenotypic assays remain the gold standard for validating resistance expression, identifying novel mechanisms, and investigating adaptive responses like efflux pump induction (Bearson et al., 2025). Isolate-based studies also enable direct assessment of sub-inhibitory effects on bacterial growth and stress physiology, which are undetectable by sequence-based analysis alone (Kneis et al., 2025).

A persistent limitation in AMR research is the compartmentalization of these methodologies. Clinical AMR studies predominantly employ strain-based phenotypic approaches, whereas environmental assessments favor metagenomics. This disconnect hinders direct, mechanistic comparisons between clinical and environmental resistance pools, limiting an integrated understanding of cross-selection across the One Health continuum.

Our study addresses this methodological gap by applying an integrated, strain-based approach to simultaneously evaluate clinical MDR isolates and environmental bacteria under identical glyphosate exposure conditions. This design enables direct phenotypic and genomic comparisons across the clinical-environmental interface, providing a mechanistic assessment of glyphosate’s potential to co-select for antibiotic resistance. We aimed to: (1) compare bacterial growth responses to glyphosate and a glyphosate-based herbicide in environmental and clinical MDR isolates from Argentina; (2) determine the antibiotic susceptibility profiles of these strains; (3) investigate the genomic basis of glyphosate resistance, with a focus on target-site modification, efflux pumps, and inactivating enzymes.

## 2 Materials and methods

### 2.1 Bacterial strains and antibiotic susceptibility

The bacterial strains and their main characteristics are summarized in Supplementary Table S1. Our bacterial collection comprised environmental strains (*n* = 68), which were randomly isolated without the application of selective pressure (antibiotics or glyphosate) from the Paraná Delta (PD) in Argentina. The remaining 35 strains were isolated from Argentinian sources and were obtained from the laboratory’s collection.

The environmental strains from the PD were isolated from wetland sediment samples taken from a Ramsar site (34.22° S, 58.89° W) during two sampling campaigns in November 2018 and December 2020. Lysogeny-broth (LB) agar and Eosin Methylene Blue (EMB) agar were used in the first and second campaigns, respectively. Colonies on plates were randomly selected and purified using the streaking technique. This technique is designed to dilute the initial microbial population, enabling the growth of well-isolated, single colonies from which pure strains can be selected for further characterization.

This study included 35 strains selected from the laboratory’s collection. Nineteen of these strains were nosocomial MDR or extensively drug-resistant isolates of the following species, which are of importance in the local clinical context: *Acinetobacter baumannii*, *Enterobacter cloacae* complex, *Escherichia coli*, *Klebsiella oxytoca*, *Klebsiella pneumoniae*, *Serratia marcescens*, and *Staphylococcus aureus*. Some of the isolates were high-risk clones (de Lagar et al., 2021) belonging to the ESKAPEE group, which stands for *Enterococcus faecium*, *Staphylococcus aureus*, *Klebsiella pneumoniae*, *Acinetobacter baumannii*, *Pseudomonas aeruginosa*, *Enterobacter* spp., and *Escherichia coli* (Krul et al., 2025). The selected clones were *A. baumannii* Global Clone 1, *K. pneumoniae* ST11 and ST258, *E. coli* ST131, and *S. aureus* ST8. The remaining 15 strains consisted of four from the laboratory’s collection, isolated from feedlots (*n* = 2), a wild animal (*n* = 1), and the environment (*n* = 1), and 11 strains isolated from herbicide-impacted soil.

Antibiotic susceptibility was tested using the disk diffusion test with Mueller-Hinton agar and antibiotic disks (Britania, Argentina). The following antibiotics were tested: ampicillin + sulbactam; aztreonam; cefalotin; ceftazidime + clavulanic acid; ceftriaxone; chloramphenicol; ciprofloxacin; colistin; erythromycin; fosfomycin; gentamicin; meropenem; tigecycline; sulfamethoxazole-trimethoprim; tetracycline; and vancomycin (for Gram-positive bacteria only). The results were interpreted according to CLSI guidelines (Lewis et al., 2023). According to Magiorakos et al. (2012), strains were categorized as having low (L), medium (M), or high (H) resistance if they were resistant to fewer than three, three, or more than three categories, respectively.

Bacterial isolates from the PD were identified to the genus level by Sanger sequencing of amplified 16S rRNA gene fragments. For DNA extraction, a single colony was subjected to thermal lysis. The colony was resuspended in 100 μL of molecular biology-grade water, incubated at 100 °C for 15 min in a dry bath (DLAB, China), and clarified by centrifugation at 10,000 × g for 10 min (DLAB, China). The resulting supernatant containing the DNA was used as template. PCR was conducted with the universal primers FD2 (AGAGTTTGATCATGGCTCAG) and RP2 (ACGGCTACCTTGTTACGACTT; Weisburg et al., 1991) using the enzyme GoTaq® from Promega (Wisconsin, USA). The PCR products were purified and sequenced by Macrogen (South Korea). For the laboratory collection strains, the 16S rRNA gene sequence was extracted from the respective whole genome sequence (WGS, Section 2.3). Taxonomic identification of the sequenced strains (except those belonging to *Enterobacter*) was carried out at the species level using Kraken2 (Wood et al., 2019). *Enterobacter* strains were identified using average nucleotide identity (ANI; Jain et al., 2018), with *Enterobacter*-type strains downloaded from the NCBI database based on the work of Wu et al. (2020).

### 2.2 Determination of minimum inhibitory concentration to glyphosate

The microplate method was used, based on the work of Bote et al. (2019). Solutions containing glyphosate or the glyphosate-based herbicide (GBH) were freshly prepared by adding the solutes to Mueller-Hinton broth (Britania, Argentina). To determine the Minimum Inhibitory Concentration (MIC) of a GBH (MIC_GBH_), we used SniperDry (Monsanto, Missouri, USA), which is a soluble concentrated solution containing 79.2 g of glyphosate monoammonium in 100 g of formulation, which corresponds to 72 g of acid equivalent (ac. eq.). In herbicide formulations, the term ac. eq. refers to the theoretical mass of the parent acid (glyphosate, or N-(phosphonomethyl)glycine), present in a given quantity of the formulated product. This is because glyphosate is often formulated as a salt (e.g., isopropylamine, potassium or ammonium) to improve solubility and ease of handling. The acid equivalent standardizes the expression of application rates based on the active herbicidal component, thereby enabling valid comparisons of efficacy and dosage across different formulations.

To measure the MIC of pure glyphosate, we used an analytical standard (Sigma-Aldrich, Massachusetts, USA). As both the herbicide and glyphosate alone acidify the medium, the pH was adjusted by adding NaOH to 6, a typical value for Argentinian agricultural soils (Alvarez et al., 2020) as measured using pH strips (DF, China). *Escherichia coli* ATCC 25922 was tested in every microplate to control the solution, which was consistently prepared in concentrations ranging from 1.12 mg/mL to 80 mg/mL ac. eq. The microplates were incubated at 25 °C overnight, and bacterial growth was estimated measuring the optical density (OD) at 630 nm using a microplate reader (Rayto RT-6000, China).

The highest concentration tested was based on the MIC of 80 mg/mL previously determined for *E. coli* (Bote et al., 2019). Although the concentrations tested in our study are higher than the manufacturers’ recommendations for field applications (10 mg ac. eq./kg), concentrations of 66.38 mg/kg have been detected in Brazilian forest soil (Da Silva et al., 2021).

### 2.3 Whole genome sequencing

Twelve PD isolates belonging to seven genera with different susceptibility to glyphosate were chosen for WGS to investigate the genomic basis of phenotypic observations and identify AMR determinants. Genomic DNA was extracted using the QIAamp® DNA Mini QIAcube Kit, and sequencing libraries were prepared using the COVIDSeq Test (Illumina, San Diego, CA, USA). The libraries were sequenced at the Malbrán Institute in Argentina using an Illumina MiSeq-I (Illumina, San Diego, CA, USA) and a MiSeq Reagent Kit v2 cartridge. The quality of the reads (2×150 bp) was inspected using FASTQC v0.11.9 (Wingett and Andrews, 2018) and adapter clipping and trimming of low-quality reads were performed using Trimmomatic v0.39 (Bolger et al., 2014). SPAdes v3.15.3 (Prjibelski et al., 2020) was used for genome assembly, and QUAST v5.0.2 for quality assessment (Mikheenko et al., 2018). Additionally, WGS data were available for 35 isolates from our laboratory’s collection (Supplementary Table S1). We used Prokka v1.14.5 (Seemann, 2014) for genome annotation. We searched for ARGs using CARD (Alcock et al., 2020), setting it to return only strict or perfect hits. Plasmids were searched using the web tool PlasmidFinder^1^ and integrons were searched using IntegronFinder (Néron et al., 2022). Among the inactivating enzymes of all the possible of the *phn* pathway targets, *phnJ* was chosen to build the phylogenetic tree because it is the key enzyme in the pathway (Morales et al., 2020).

### 2.4 Graphics and data analysis

Maximum likelihood (ML) phylogenetic trees were constructed using R (v4.5.1) with the ape, phangorn, Biostrings, and ggtree packages (R Foundation for Statistical Computing, 2025) for the 16S rRNA gene (nucleotide sequences) and for the *aroA* and *phnJ* genes (amino acid sequences). For the 16S rRNA analysis, the sequences were aligned using the SILVA database reference alignment (Glöckner et al., 2017). The best-fit model of evolution was selected for protein-coding genes using the Akaike Information Criterion, identifying WAG for *aroA* (AIC: 18,480.56) and LG for *phnJ* (AIC: 3,415.18). The Jukes-Cantor model was used for 16S rRNA. An initial Neighbor-Joining tree was constructed for all genes and subsequently optimized under an ML framework with stochastic rearrangement. Node support was assessed through 1000 bootstrap replicates with topology optimization for each replicate. The figures were generated using *ggplot2* (Alboukadel, 2018) and *ggtree* (Yu et al., 2017), and were subsequently edited in Inkscape.^2^

### 2.5 Data availability statement

The raw data on bacterial growth is available at 10.6084/m9.figshare.29696513 (pure glyphosate) and 10.6084/m9.figshare.29696861 (GBH). The 16S rRNA gene sequences are available at 10.6084/m9.figshare.29695523. Whole genome sequencing data can be found in GenBank under the accession numbers PRJNA1298335 for PD strains and PRJNA1357289 for herbicide-impacted soil strains.

## 3 Results

### 3.1 A glyphosate-based herbicide inhibits the growth of environmental bacteria, even at low concentrations within the tested spectrum

A total of 68 strains were isolated from sediment in a Ramsar site area at the PD, Argentina, across two sampling campaigns. Strains isolated using non-selective LB agar in the first campaign were identified as belonging to the genera *Acinetobacter* (*n* = 1), *Bacillus* (*n* = 12), *Enterobacter* (*n* = 8), *Exiguobacterium* (*n* = 1), *Lysinibacillus* (*n* = 1), *Pantoea* (*n* = 2), *Pseudomonas* (*n* = 5), and *Scandinavium* (*n* = 1). For the second campaign, EMB medium was used for its selectivity for Gram-negative bacteria, thereby promoting a greater diversity of isolates from this group. The second campaign’s strains were classified as follows: *Acinetobacter* (*n* = 4), *Burkholderia* (*n* = 4), *Chryseobacterium* (*n* = 3), *Comamonas* (*n* = 1), *Elizabethkingia* (*n* = 2), *Enterobacter* (*n* = 2), *Ochrobactrum* (*n* = 1), *Pseudomonas* (*n* = 4), *Sphingobacterium* (*n* = 2), and *Stenotrophomonas* (*n* = 14).

**Figure 2:**
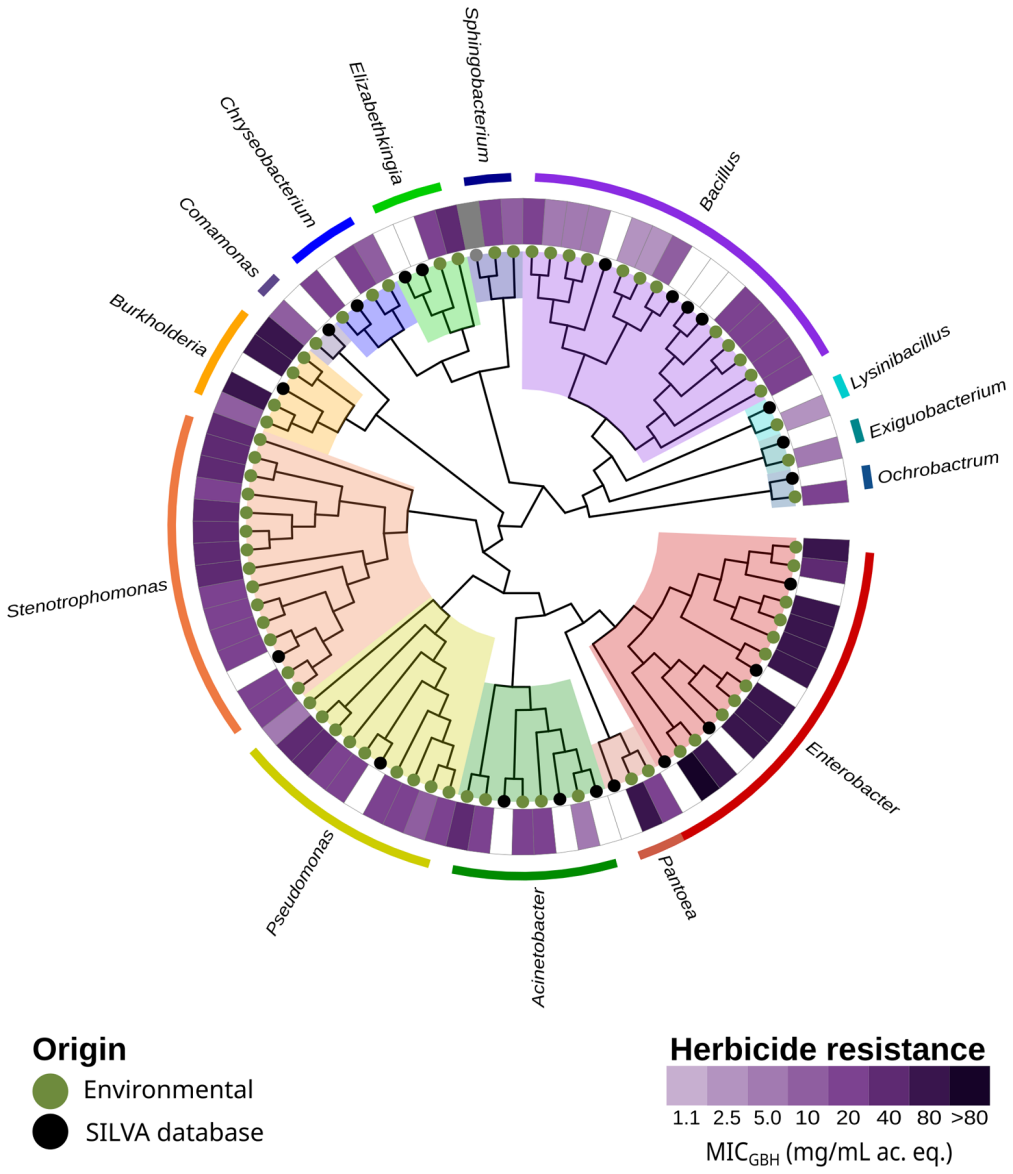
Phylogenetic tree of the 16S rRNA gene of the Paraná Delta environmental strains. The ring shows the minimum inhibitory concentration to a glyphosate-based herbicide. The circle’s colors at the tipping point of the branches indicate the isolate’s origin. The tree was built using the SILVA alignment tool and ggtree in R. MIC_GBH_, Minimum inhibitory concentration to a glyphosate-based herbicide. Ac. eq., acid equivalent.

To test their resistance to GBH and pure glyphosate, we conducted the tests after establishing a pH value of 6 to simulate agricultural conditions. The MIC of the 68 strains ranged between 2.5 and >80 mg/mL in both cases (Figure 2). Strains of the genus *Enterobacter*, which play a critical role in the local hospital environment (Echegorry et al., 2024), tolerated the highest concentrations. Strains of the genus *Bacillus* were particularly susceptible to the presence of the herbicide.

**Figure 3:**
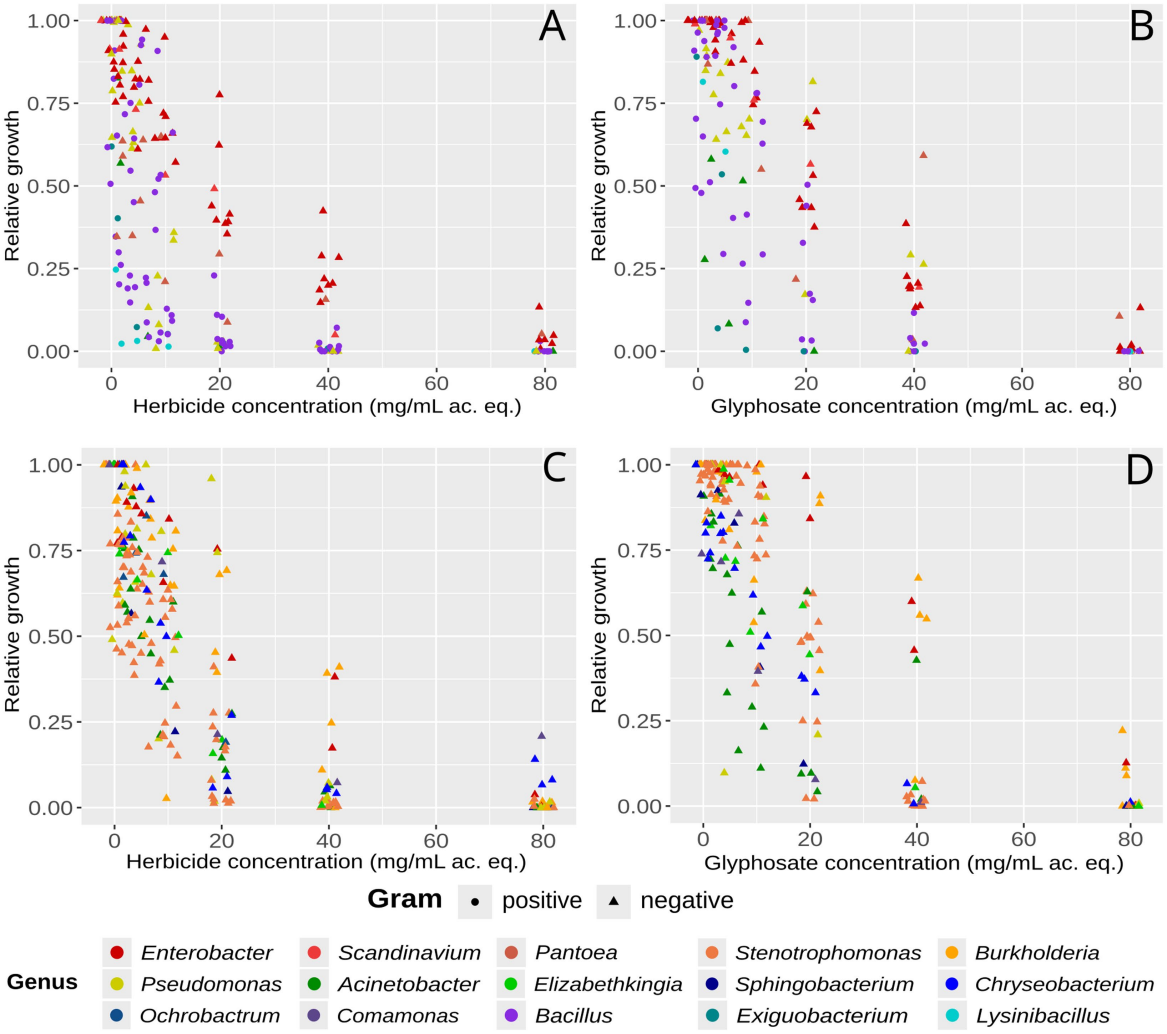
Effect of a glyphosate-based herbicide **(A,C)** and pure glyphosate **(B,D)** on bacterial growth. Figures **A,B** correspond to the first campaign at the Paraná Delta (LB medium) and **C,D** to the second campaign (EMB medium). Growth was estimated by measuring optical density, and results were standardized to the growth of the same strain in the absence of glyphosate. The figure was built using the ggplot2 package in R. Ac. eq., acid equivalent.

Increasing concentrations of GBH or pure glyphosate (Figure 3) inhibited growth for most isolates, even at the lowest concentration tested (1.12 mg/mL eq. ac.). Although glyphosate contributed partially to the growth inhibition in all cases, the effect was significantly greater with GBH, by a factor of one to four dilutions.

### 3.2 Environmental and clinical isolates share common genera with high glyphosate resistance

**Figure 4:**
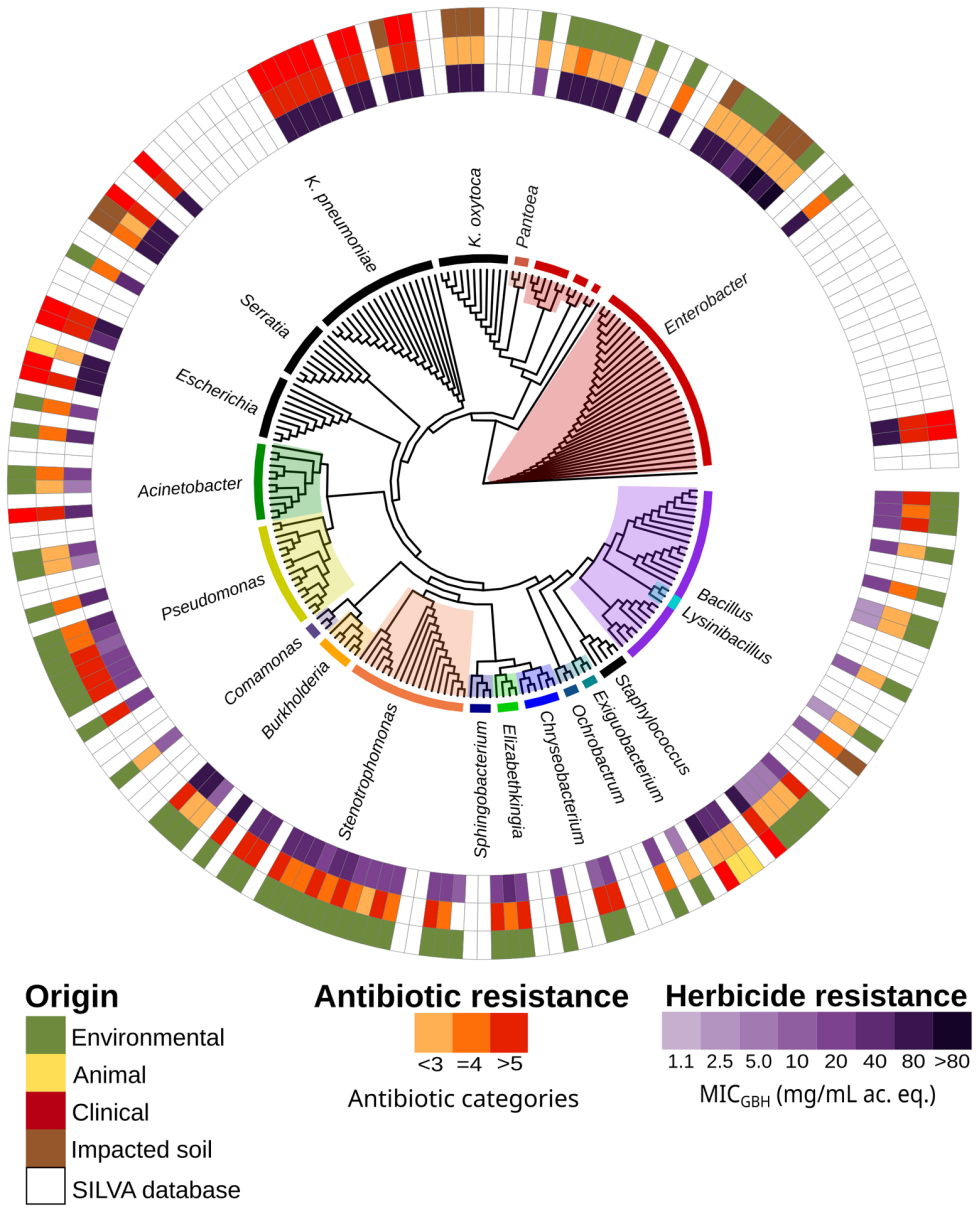
Phylogenetic tree of the 16S rRNA gene of all strains used in this study. The outer ring shows the origin of the isolates. The middle ring shows phenotypic resistance to antibiotics. The innermost ring shows the minimum inhibitory concentration to a glyphosate-based herbicide. The tree was built using the SILVA alignment tool and ggtree package in R. MIC_GBH_, minimum inhibitory concentration to a glyphosate-based herbicide. ac. eq., acid equivalent.

To investigate the relationship between glyphosate resistance and clinical MDR, an additional 35 isolates from our laboratory collection were analyzed, alongside the 68 strains from the PD site. (n_total_ =103, Supplementary Table S1). Regarding AMR phenotypes (Supplementary Table S2), no pattern was observed that correlated this trait with glyphosate resistance in all strains (Supplementary Table S3). The *p*-value of the Spearman correlation was Rho = 0.003, and *p* = 0.977. In contrast, clinical MDR strains, which are commonly associated with hospital-acquired infections (Macesic et al., 2025) were found to be resistant to glyphosate (Figure 4). The median MIC_GBH_ value was 20 mg/mL for PD strains and 80 mg/mL for clinical strains. A 16S rRNA gene phylogenetic tree (Figure 4) showed that environmental strains exhibiting high resistance to GBH clustered closely with clinical MDR strains. Strains belonging to the genus *Enterobacter* predominated among glyphosate-resistant isolates exhibiting this relationship. One of the environmental *Enterobacter* strains (B2) was identified as *E. kobei*, a species belonging to the *Enterobacter cloacae* complex which showed the highest MIC_GBH_ of all the PD strains in this study.

Table 1 presents a summary of the clinical strains and their main characteristics. The 19 clinical isolates from Argentina belonged to the following species: *Acinetobacter baumannii* (*n* = 1), *E. coli* (*n* = 3), *E. hormaechei* (*n* = 2), *K. oxytoca* (*n* = 2), *K. pneumoniae* (*n* = 7), and *S. marcescens* (*n* = 2). *Staphylococcus aureus* (*n* = 2) was the only Gram-positive representative of the nosocomial niche. Fourteen of the isolates were resistant to carbapenems: 12 had *bla*_KPC-2_, one had *bla*_NDM-5_, one had *bla*_OXA-163_, and one had *mecA* (in the case of *S. aureus*). Eight of the isolates were high-risk clones.

**Table 1:** Clinical strains analyzed in this study (*n* = 20).

Strain	Species	ST	High risk clone	Resistance	Reference
A144	*Acinetobacter baumannii*	GC1	Yes	AMS, CAZ, CIP, CTX, GEN, LEV, STX, TET	(Álvarez et al., 2020)
HA4Ec	*Escherichia coli*	730	No	AM, AMC, CAZ, CZ, CRO, ETP, FEP, IMI, MEM, PTZ	(Álvarez et al., 2023)
HA25Ec	*Escherichia coli*	648	No	CAZ, CIP, CRO, ETP, FEP, IMP, MEM, MIN, PTZ, SXT	(Piekar et al., 2023)
SM5_ST131	*Escherichia coli*	131	Yes	AMC, AMN, AMS, AZT, CAZ, CIP, CRO, FEP, FOS, TAZ, TMS	(Carrera Páez et al., 2024)
BA_SM_C105	*Serratia marcescens*	-	-	AMS, CEF, COL, ERY	-
ER279	*Serratia marcescens*	-	-	AMK, AZT, CTX, ERY, FEP, GEN	-
HA16Eho	*Enterobacter hormaechei*	-	-	CAZ, CIP, CTX, FEP, IMP, MEM, NFT, NOR, SXT, TZP	(Knecht et al., 2022a)
HA2pEho	*Enterobacter hormaechei*	45	No	AMS, CRO, CAZ, FEP, IMP, MEM, ETP, CAZ/AVI, SXT, TAZ, CIP, NFT	(Knecht et al., 2022b)
HA7pKpn	*Klebsiella pneumoniae*	18	No	AMS, CRO, CAZ, FEP, IMP, MEM, ETP, CAZ/AVI, SXT, TAZ	(Knecht et al., 2022b)
HA31Kpn	*Klebsiella pneumoniae*	11	Yes	AMK, CIP, CRO, GEN, IMI, MEM, PTZ, SXT	(Álvarez et al., 2024)
HA39pKp	*Klebsiella pneumoniae*	11	Yes	AMS, CAZ, CIP, CL, CRO, ETP, FEP, IMI, MEM, NFT, PTZ, SXT	(Allende et al., 2024)
HA3pKpn	*Klebsiella pneumoniae*	258	Yes	AM, AMC, AMK, CAZ, CIP, CRO, CZ, ETP, FEP, FOS, GEN, IMI, MEM, NFT, PTZ, SXT	(Álvarez et al., 2023)
KpS26	*Klebsiella pneumoniae*	15	No	AZT, CAZ, CDZ, CFX, CIP, CMP, COL, CTX, ETP, FEP, IMI, MEM, PTZ, STR, SXT, TET	(Álvarez et al., 2022)
HA40pKp	*Klebsiella pneumoniae*	11	Yes	AMS, AN, CAZ, CIP, CL, CRO, ETP, FEP, NFT, IMI, MEM, MIN, PTZ	(Allende et al., 2024)
HA49pKp	*Klebsiella pneumoniae*	11	Yes	AM/AMC/CZ, CAZ, CIP, CL, CRO, ETP, FEP, GEN, IMI, MEM, NFT, PTZ, SXT	(Allende et al., 2024)
HA10pKo	*Klebsiella oxytoca*	2	No	AMS, CAZ, CIP, CRO, ETP, GEN, IMI, MEM, MIN, PTZ, TIG, SXT	(Álvarez et al., 2025)
HA8pKo	*Klebsiella oxytoca*	2	No	AMS, CRO, CAZ, CIP, ETP, GEN, IMI, MEM, MIN, PTZ, SXT	(Álvarez et al., 2025)
HU14	*Staphylococcus aureus*	5	No	AMS, AZT, CAZ/CLV, CEF, CIP, CRO, ERY, GEN, MEM	(Lattar et al., 2012)
HU78	*Staphylococcus aureus*	8	Yes	AZT	(Lattar et al., 2012)

19 were isolated in Argentina and one is a type strain used as a control. AM, ampicillin; AMC, amoxicillin-clavulanic acid; AMS, amoxicillin-sulbactam; AMK, amikacin; AZT, aztreonam; CDZ, cadazolid; CFX, cefoxitin; CMP, chloramphenicol; CL, clindamycin; CTX, cefotaxime; CAZ, ceftazidime; CAZ/CLV, ceftazidime-clavulanic acid; CZ, cefazolin; CIP, ciprofloxacin; COL, colistin; CRO, ceftriaxone; ERY, erythromycin; ETP, ertapenem; FEP, cefepime; GEN, gentamicin; IMI, imipenem; MEM, meropenem; MIN, minocycline; NFT, nitrofurantoin; NOR, norfloxacin; PTZ, piperacillin-tazobactam; STR, streptomycin; SXT, sulfametoxazole-trimethoprim; TET, tetracyclin; and TIG, tigecycline GC1, Global Clone 1.

### 3.3 Genome sequencing revealed several mechanisms of herbicide resistance

Whole genome sequencing (WGS) data from 12 PD isolates and 34 laboratory collection strains belonging to the genera *Acinetobacter* (*n* = 3), *Enterobacter* (*n* = 9), *Escherichia* (*n* = 4), *Klebsiella* (*n* = 13), *Pantoea* (*n* = 1), *Pseudomonas* (*n* = 3), *Serratia* (*n* = 5), *Rossellomorea* (*n* = 1, formerly included in the genus *Bacillus*), *Priestia* (*n* = 1), *Scandinavium* (*n* = 1, formerly included in the genus *Enterobacter*), *Staphylococcus* (*n* = 4), and *Stenotrophomonas* (*n* = 1) were analyzed. In addition to efflux pumps, several ARGs with other AMR mechanisms, including beta-lactamases (classes A, B3, C, and D), were identified in the PD strains. Regarding mobile genetic elements (MGEs), no PD strains were found to carry integrons, and only three carried plasmids (Supplementary Table S4). Two of these were *Enterobacter*, and the third was identified as *Pantoea agglomerans*. Of the two *Enterobacter* strains, the assembled plasmid pB2 from *E. kobei* B2 was 113,038 bp long and belonged to the incompatibility group IncFIB. The Li2LvB3 strain was classified as *E. soli* and its plasmid (pLi2LvB3) of 86,882 bp belonged to the IncFII incompatibility group. Complete, circular assembly using Illumina short-read alone is challenging, as repetitive regions can prevent closure. Therefore, the reported sizes are likely to be underestimates. The *P. agglomerans* strain carried a ~ 622 kb megaplasmid belonging to the Large *Pantoea* Plasmids (LPP-1), which are common to all *Pantoea* species (De Maayer et al., 2012). In this case, the megaplasmid harbors ARGs which code for multidrug-resistance efflux pumps (*acrBE*).

**Figure 5:**
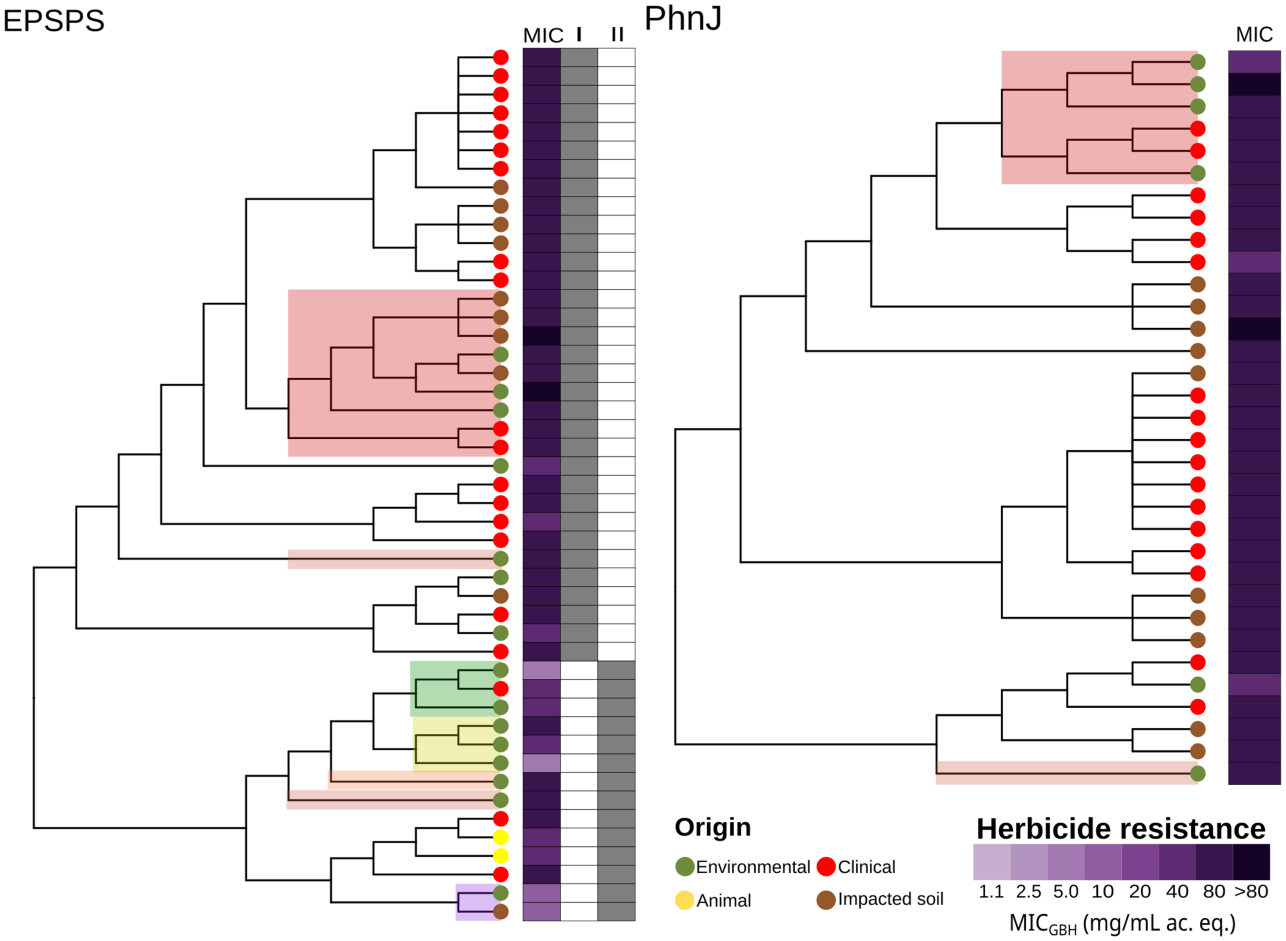
Phylogenetic trees of the translated sequences of *aroA* (EPSPS) and *phnJ* (PhnJ). The heatmap on the right of the tree shows the minimum inhibitory concentration to a glyphosate-based herbicide and the EPSPS class (I and II) assessed with the online tool: http://ppuigbo.me/programs/EPSPSClass/. MIC_GBH_: minimum inhibitory concentration to a glyphosate-based herbicide. Ac. eq.: acid equivalent.

**Figure 6:**
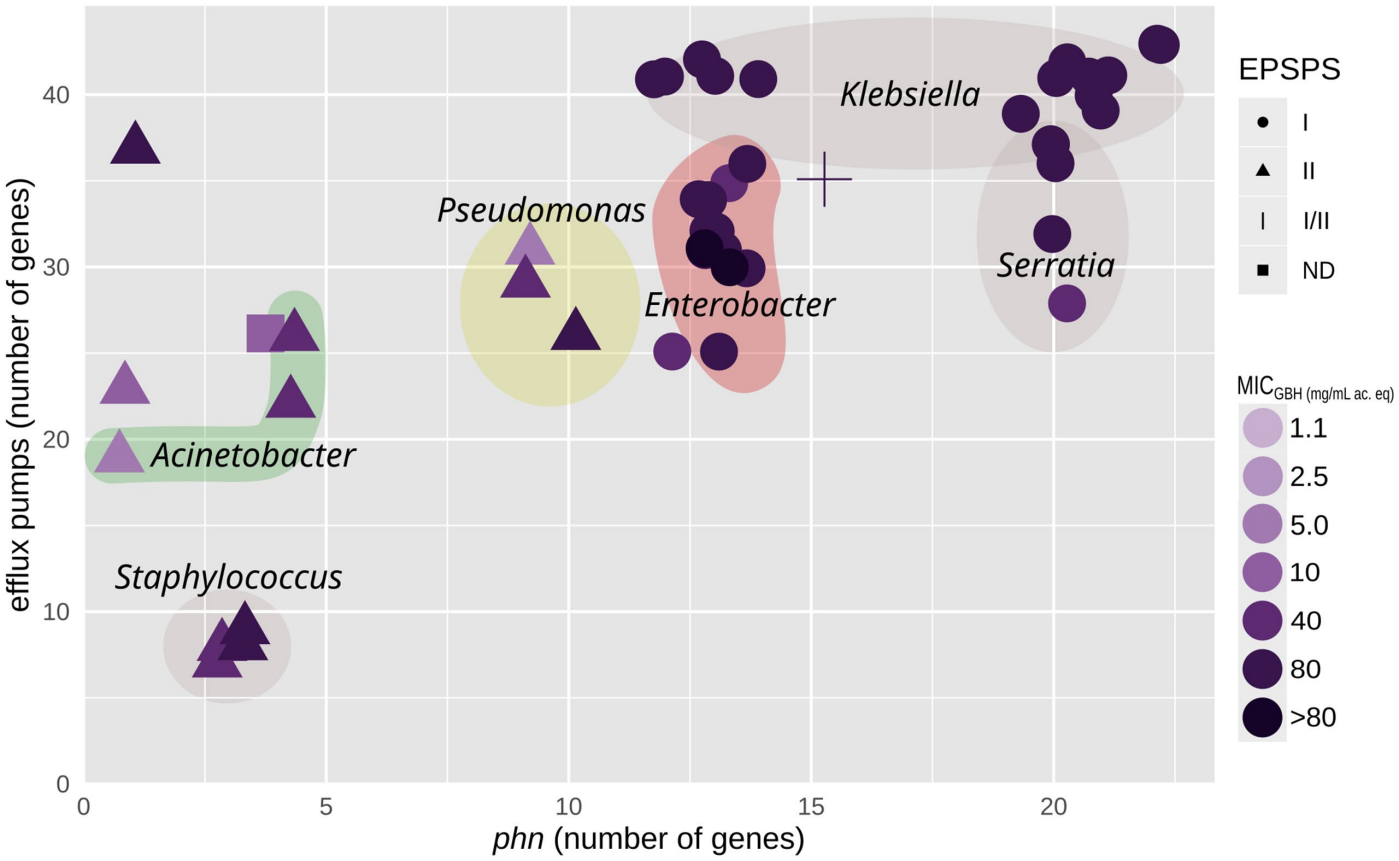
Scatter plot of whole genome sequence of 46 strains. The number of genes in the *phn* cluster is shown on the *x*-axis, and the number of genes that code for antimicrobial efflux pumps is shown on the *y*-axis. The shape indicates the EPSPS type, and shade tone of violet shows the minimum inhibitory concentration to the glyphosate-based herbicide. This figure was created using the ggplot2 package in R. MIC_GBH_, minimum inhibitory concentration to a glyphosate-based herbicide. Ac. eq., acid equivalent.

Figure 5 shows the phylogenetic trees of the *in silico*-translated EPSPS and PhnJ sequences. The EPSPS sequences are distributed across classes I and II. Strains with class I EPSPS were generally more resistant to glyphosate than those with class II EPSPS. The three previously described glyphosate resistance mechanisms were analyzed: (1) target site modification (EPSPS classes II, III, and IV *aroA* genes), (2) inactivating enzymes (*phn* genes), and (3) efflux pumps, such as *yhhS* (Staub et al., 2012), *mexAB* (da Barbosa Costa et al., 2022), and *acrAB* (Singh et al., 2022). Figure 6 shows a correlation analysis between MIC_GBH_ and AMR determinants, including EPSPS classes and the number of *phn* and efflux pump genes. The *phnJ* gene was detected in approximately half of the analyzed genomes. We identified efflux pump genes from the RND, SMR, MFS, MATE, and ABC families, with the RND-type systems MexAB and AcrAB being particularly noteworthy due to their documented links to GBH stress (Pöppe et al., 2020; da Barbosa Costa et al., 2022; Singh et al., 2022). MexAB system genes increase under GBH stress (da Barbosa Costa et al., 2022) and were ubiquitous in *Pantoea*, *Klebsiella*, and *Stenotrophomonas* isolates. They were variably present in *Enterobacter*, *Acinetobacter*, *Serratia*, *Escherichia*, and *Pseudomonas* isolates and absent in *Scandinavium*, *Rossellomorea*, and *Staphylococcus* isolates. In contrast, the *acrAB* genes, which are directly linked to glyphosate adaptation and associated with AMR (Pöppe et al., 2020; Singh et al., 2022), were present in all genera except for *Pseudomonas*, *Rossellomorea*, and *Staphylococcus aureus*. Overall, the number of efflux pumps and *phn* genes appeared to be a more critical factor in resistance than the EPSPS class.

## 4 Discussion

The objective of our study was to analyze bacterial resistance to both GBH, pure glyphosate, and antibiotics in environmental and clinical isolates from Argentina, a country that uses these herbicides extensively (National Registry of Plant Therapeutics of SENASA, 2023). We evaluated the effect of a GBH and glyphosate alone on bacterial growth at a pH close to neutrality. A clear glyphosate resistance gradient was observed among environmental bacteria from a protected Ramsar site in the PD, a location not directly exposed to herbicides. In contrast, all tested clinical strains exhibited high resistance. Within the environmental strains gradient, those at the upper limit were phylogenetically related to clinical pathogens. Initial screening revealed that even the lowest tested concentrations of GBH inhibited the growth of PD environmental isolates, with the extent of inhibition depending on the genus. By repeating the test but with only glyphosate, we observed that part of this effect was exerted by the herbicide’s active compound, although the inhibitory effect was weaker in this case.

While there is currently no consensus on whether glyphosate use can significantly affect microbial community composition, evidence suggests that it can favor pathogenic and resistant traits (Motta et al., 2018; Liao et al., 2021). Glyphosate disturbs honeybee gut microbiota, resulting in a decrease in the abundance of key bacterial species and a subsequent reduction in the host’s resistance to pathogens (Motta et al., 2018). Many of the genera found in this study have been reported to be able to degrade glyphosate (Guijarro et al., 2018; Singh et al., 2019; Firdous et al., 2020; Aso et al., 2021; Hertel et al., 2021; Masotti et al., 2021; Zhang et al., 2022). While metabolizing the herbicide, the sustained exposure may concurrently select for traits that enhance clinical risk. Among these genera in our study, isolates belonging to *Pseudomonas* and *Burkholderia* demonstrated glyphosate and AMR. This finding has potential implications for human health because both play a key role in the environmental fate of glyphosate and include opportunistic pathogens (Berg et al., 2005; Castrejón-Godínez et al., 2021; Sibalekile et al., 2025). Members of the genus *Enterobacter* were frequently among the most glyphosate-resistant environmental isolates, including one *E. kobei* strain that is part of the *Enterobacter cloacae* complex. This is consistent with previous findings (Gao et al., 2021), which showed that exposure to 5 mg/L and 10 mg/L of glyphosate can significantly increase the copy number of *Enterobacter* spp. in a beetle’s microbiome after 5 days. A recent study from Argentina (Cuzziol Boccioni et al., 2023) found that *Enterobacter* was enriched in tadpole gut microbiomes after exposure to GBH in a concentration of 2.5 mg/L. It is particularly concerning that glyphosate can enrich for *Enterobacter,* given that this genus is an emerging pathogen in Argentina (Knecht et al., 2022b) and can exhibit multidrug-resistance in the environment (Ghiglione et al., 2021). Studies focusing on polluted environments have identified agricultural sites as significant reservoirs for ARGs (Bearson et al., 2025). In these areas, the application of animal manure and commercial herbicides like glyphosate acts as a primary driver for the enrichment of MGEs and ARGs within soil microbiomes. Liao et al. (2021) found that glyphosate exposure increases the prevalence of ARGs and MGEs in soil microbiomes. This was demonstrated by a ninefold increase in total ARG abundance in microcosm experiments over 60 days, as well as by promoting the horizontal genetic transfer (HGT) of resistance plasmids between bacteria. Research indicates that glyphosate exposure can escalate HGT by increasing cell membrane permeability, thereby stimulating the conjugative transfer of multidrug-resistance plasmids (Li et al., 2022). Specifically, broad host range plasmids of the IncP-1 group, which are frequently associated with both antibiotic resistance and catabolic genes, have been exclusively detected in the rhizosphere of glyphosate-treated plants and prevalent in on-farm biopurification systems (Allegrini et al., 2019). The proliferation of these plasmids, particularly the IncP-1ε subgroup, is recognized as a major risk for the dissemination of clinical resistance across agro-ecosystems (Zabaloy et al., 2022). The *Bacillus* isolates in our study showed the highest susceptibility to glyphosate. This is particularly relevant in agriculture because some *Bacillus* species are used as biopesticides (Palma et al., 2024) and growth promoters in animal production (Golnari et al., 2024).

For the clinical niche, we selected MDR strains that were representative of species that are relevant in the local context. Most of these strains were part of the ESKAPEE group (Krul et al., 2025), with some being high-risk clones. In addition to *E. hormaechei*, strains belonging to *K. pneumoniae*, *K. oxytoca*, *E. coli*, *S. marcescens*, and *S. aureus* exhibited high levels of resistance to GBH. A recent study on Gram-negative pathogens revealed a synergistic effect between GBH and carbapenems (Zerrouki et al., 2024). The work proposed that this synergy occurs through two mechanisms: the alteration of outer membrane permeability and the chelation of metals in metallo-beta-lactamases. The MIC_GBH_ values obtained in our study were higher than those described in previous studies for clinical isolates (Zerrouki et al., 2024), probably because the pH was not neutralized in those cases. Another study (Háhn et al., 2022) showed that sub-lethal glyphosate exposure can induce specific antibiotic resistance in *P. aeruginosa*, such as to the last-resort carbapenem imipenem, potentially via regulation of the *oprD* porin. Some strains of *S. aureus* have previously been found to be resistant to glyphosate (Priestman et al., 2005).

**Figure 7:**
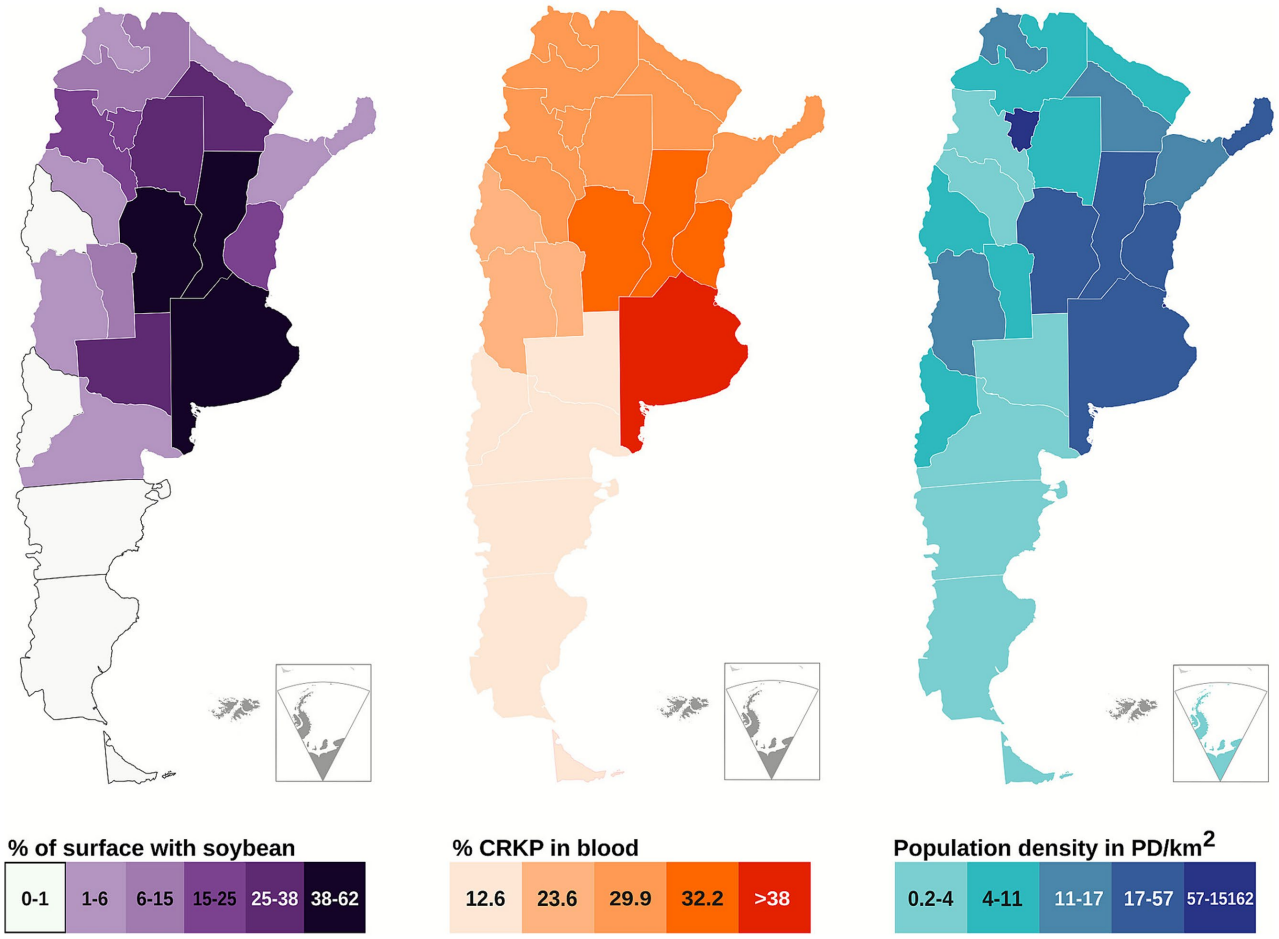
Maps of the co-occurrence of MDR strains and exposure to glyphosate. Soybean cultivation is shown as a percentage of each province’s total area, CRKP as a percentage of positive isolates in the region, and population density as the number of people per square meter. The data covers the period from 2022 to 2024 and has been adapted from the following sources: Instituto de Estadísticas y Censos (INDEC, 2022, https://www.indec.gob.ar/); Red WHONET Argentina (2024); and Instituto Nacional de Semillas (https://www.argentina.gob.ar/inase, 2024). CRKP: carbapenem-resistant *Klebsiella pneumoniae*. The depiction of boundaries on this map is based on the source data from Argentine institutions and is for illustrative purposes only. The inclusion of the Islas Malvinas, Georgias del Sur, and Sandwich del Sur, and the Argentine claim in Antarctica does not imply an official position by Germany, which remains neutral on these territorial matters.

Figure 7 shows an analysis of the geographical distribution of the risk of co-occurrence of MDR strains and exposure to glyphosate, as well as carbapenem-resistant *K. pneumoniae* isolated from blood, soybean cultivation, and population density. According to the 2023 report from the Argentine AMR surveillance system,^3^ carbapenem resistance is a critical issue. The proportion of isolates resistant to carbapenems that year was: *A. baumannii* (86.8%, *n* = 2,178), *K. pneumoniae* (35.6%, *n* = 7,947), *Enterobacter* spp. (12.3%, *n* = 2,038), and *E. coli* (2.3%, *n* = 4,837). In the case of *Staphylococcus aureus*, 24% of the isolates were methicillin-resistant (*n* = 7,525). All soybean cultivation in Argentina involves genetically modified plants, most of which are resistant to glyphosate (Yankelevich, 2024), implying that GBH use is concomitant with soybean cultivation. The most densely populated region overlaps with the region where most soybean cultivation is currently taking place. Therefore, the GBH selective pressure exerted by agricultural soils represents a potential selective force for clinical bacteria that usually enter the environment through wastewater (Endalamaw et al., 2024). In addition to the selective pressure exerted by GBH in agricultural soils, other niches should be considered, such as horticulture (Mac Loughlin et al., 2017), food (Rawat et al., 2023), and water (Mac Loughlin et al., 2022). As the trajectory of GBH or glyphosate is not well understood, several niches could act as reservoirs and sites for the selection of clinical strains.

Most glyphosate-resistant strains found in the environment were phylogenetically related to MDR clinical strains. As previously suggested by da Barbosa Costa et al. (2022) and confirmed by our study, this could be due to cross-selection via efflux pumps. Our genomic comparison of environmental and clinical strains (Figure 6) supports that hypothesis, as it generally showed a higher number of efflux pumps in those isolates that were more resistant to the herbicide. Genomic analysis (Figures 5, 6) also revealed that *phn* genes and efflux pumps were more relevant than the EPSPS class in enabling bacterial growth in the presence of glyphosate. This confirms the findings of Masotti et al. (2021) and Motta et al. (2018).

## 5 Conclusions and future perspectives

Our results further highlights the links between clinically relevant species and herbicide use in agricultural soils. We identified a resistance gradient in environmental isolates from a protected wetland, with the most resistant strains being phylogenetically related to MDR clinical pathogens. Genomic evidence indicates that mechanisms such as efflux pumps and degradative genes are key drivers of this phenotype, facilitating the co-selection of resistance traits. Our geographical risk analysis further underscores the concerning overlaps between regions of intensive soybean cultivation and its associated GBH use, areas of high population density and consequent wastewater production, and the prevalence of critical clinical infections, such as carbapenem-resistant *K. pneumoniae*. These findings collectively underscore that the selective pressure exerted by a common agrochemical could contribute to shaping a resistance landscape with direct implications for public health, reinforcing the need for a One Health approach to AMR.

Future research should employ long-read genomic surveillance to track the flow of bacterial species and MGEs carrying ARGs through critical interface niches like irrigation water, horticulture, and livestock wastewater. This genomic tracking should be coupled with the concurrent chemical quantification of microcontaminants, including glyphosate and other agrochemicals, to clarify exposure pathways and allow for a robust estimation of risks. Translating this knowledge into mitigation strategies, such as improved wastewater treatment from both human and livestock sources, is part of the actions needed to safeguard antibiotic efficacy.

## Data Availability

The datasets presented in this study can be found in online repositories. The names of the repository/repositories and accession number(s) can be found at: https://www.ncbi.nlm.nih.gov/, PRJNA1298335; https://www.ncbi.nlm.nih.gov/, PRJNA1357289.

## Footnotes

1
https://cge.food.dtu.dk/services/PlasmidFinder/

2
https://inkscape.org

3
https://antimicrobianos.com.ar/whonet-argentina/
